# *N*-acetyltaurine and Acetylcarnitine Production for the Mitochondrial Acetyl-CoA Regulation in Skeletal Muscles during Endurance Exercises

**DOI:** 10.3390/metabo11080522

**Published:** 2021-08-06

**Authors:** Teruo Miyazaki, Yuho Nakamura-Shinya, Kei Ebina, Shoichi Komine, Song-Gyu Ra, Keisuke Ishikura, Hajime Ohmori, Akira Honda

**Affiliations:** 1 Joint Research Center, Tokyo Medical University Ibaraki Medical Center, Ami, Tokyo 300-0395, Japan; akihonda@tokyo-med.ac.jp; 2 Department of Immunology, Faculty of Medicine, University of Tsukuba, Tsukuba 305-8575, Japan; yuhojump@yahoo.co.jp; 3 Department of Health and Nutrition, Tsukuba International University, Tsuchiura 300-0051, Japan; ebinakei@gmail.com; 4 Department of Acupuncture and Moxibustion, Faculty of Human Care, Teikyo Heisei University, Toshima-ku, Tokyo 170-8445, Japan; s.komine@thu.ac.jp; 5 Faculty of Medicine, University of Tsukuba, Tsukuba 305-0821, Japan; 6 Institute of Liberal Arts and Sciences, Tokushima University, Tokushima 770-8502, Japan; songgyura@tokushima-u.ac.jp; 7 Faculty of Management, Josai University, Sakado 350-0295, Japan; ishikura@josai.ac.jp; 8 Faculty of Health and Sport Sciences, University of Tsukuba, Tsukuba 305-8574, Japan; omori.hajime.gb@u.tsukuba.ac.jp; 9 Department of Gastroenterology and Hepatology, Tokyo Medical University Ibaraki Medical Center, Ami, Tokyo 300-0395, Japan

**Keywords:** taurine, NAT, carnitine, ACT, acetyl-CoA, mitochondria, endurance exercise, skeletal muscle

## Abstract

During endurance exercises, a large amount of mitochondrial acetyl-CoA is produced in skeletal muscles from lipids, and the excess acetyl-CoA suppresses the metabolic flux from glycolysis to the TCA cycle. This study evaluated the hypothesis that taurine and carnitine act as a buffer of the acetyl moiety of mitochondrial acetyl-CoA derived from the short- and long-chain fatty acids of skeletal muscles during endurance exercises. In human subjects, the serum concentrations of acetylated forms of taurine (NAT) and carnitine (ACT), which are the metabolites of acetyl-CoA buffering, significantly increased after a full marathon. In the culture medium of primary human skeletal muscle cells, NAT and ACT concentrations significantly increased when they were cultured with taurine and acetate or with carnitine and palmitic acid, respectively. The increase in the mitochondrial acetyl-CoA/free CoA ratio induced by acetate and palmitic acid was suppressed by taurine and carnitine, respectively. Elevations of NAT and ACT in the blood of humans during endurance exercises might serve the buffering of the acetyl-moiety in mitochondria by taurine and carnitine, respectively. The results suggest that blood levels of NAT and ACT indicate energy production status from fatty acids in the skeletal muscles of humans undergoing endurance exercise.

## 1. Introduction

**Figure 1:**
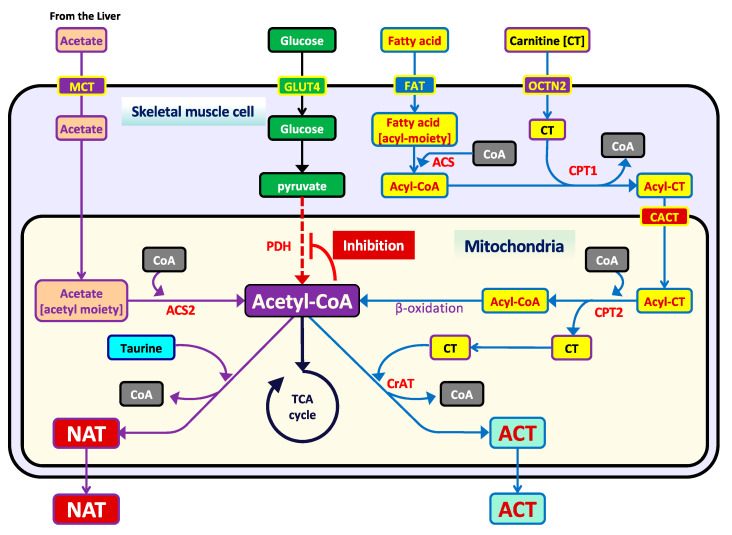
The preventive mechanisms for acetyl-CoA accumulation in the mitochondria through the acetylation of taurine and carnitine. An excess accumulation of mitochondrial acetyl-CoA metabolized from fatty acid and acetate would cause the metabolic stagnation of the glycolytic pathway as negative feedback through allosteric inhibition of PDH. To prevent the accumulation of mitochondrial acetyl-CoA, taurine and carnitine play buffering roles on the acetyl moiety of mitochondrial acetyl-CoA. Consequently, ACT and NAT are produced from fatty acids and acetate, respectively, and then excreted to extracellular fluids. Abbreviations: ACT; acetylcarnitine, ACS; acyl-CoA synthetase, ACS2; acetyl-CoA synthetase 2, CACT; carnitine acylcarnitine translocase, CPT; carnitine palmitoyl transferase, CrAT; carnitine acetyltransferase, CT; carnitine, GLUT4; glucose transporter 4, MCT; monocarboxylate transporter, NAT; *N*-acetyltaurine, OCTN2; organic anion transporter 2, PDH; pyruvate dehydrogenase.

During energy production, mitochondrial acetyl-CoA is the metabolic junction that connects the TCA cycle with the upstream metabolic pathways of nutrient energy fuels, including carbohydrates, lipids, and amino acids. During endurance exercises, large amounts of ATP are produced from the acetyl-CoA in the mitochondria of skeletal muscles to meet the increasing energy demand [1,2]. An elevation in the mitochondrial acetyl-CoA/free-CoA ratio, accompanied by an increase in acetyl-CoA production, would suppress the metabolic flux from glycolysis to the TCA cycle in the skeletal muscles, because acetyl-CoA is an allosteric inhibitor of pyruvate dehydrogenase (PDH) (Figure 1) [3]. This feedback system is the role metabolic switches play to efficiently produce energy depending on the availability of energy fuels.

In long-chain fatty acid β-oxidation, carnitine (β-hydroxy-γ-*N*-trimethylaminobutyric acid) plays important roles as an essential carrier for the transport of long-chain fatty acids from the cytoplasm into the mitochondrial matrix (Figure 1) and a physiological modulator of the mitochondrial pool of acetyl-CoA; i.e., the acetyl moiety of acetyl-CoA is converted to carnitine by carnitine acetyltransferase (CAT), which is located within the mitochondrial matrix, and, consequently, yields acetylcarnitine (ACT) and free-CoA [4]. Thereafter, ACT is excreted into the extracellular fluid. As the β-oxidation is activated for large energy production in endurance exercises, we suggest that the level of ACT in the blood increases during endurance exercises.

In addition, acetyl-CoA is produced in the mitochondria of skeletal muscles from the liver-supplied lipid metabolites, such as ketone bodies and a short-chain fatty acid, acetate. Although the utilization of ketone bodies in the skeletal muscles for energy production in endurance exercises has been well-known, acetate is also utilized as an efficient substrate for the production of mitochondrial acetyl-CoA via one metabolic reaction by acetyl-CoA synthetase 2 (ACS2) (Figure 1) [5,6,7,8]. Therefore, the acetyl-CoA derived from acetate is suggested to accumulate in the mitochondria during endurance exercises.

In a study by Shi et al. [9], the acetate yielded from ethanol detoxication in the liver reacted with taurine (2-aminoethanesulfonic acid), consequently forming *N*-acetyltaurine (NAT) for the facilitation of acetate excretion in the urine. Taurine exists in high concentrations in the whole body, particularly in the skeletal muscles and the liver [10,11,12], and it exhibits many physiological functions [13,14,15,16,17]. Taurine has been reported to enhance exercise performance in humans [18] and rodents [19,20,21], and its concentration in the skeletal muscles of rats significantly and time-dependently decreased during endurance exercises [13,14,15,16,17]. However, the reasons why taurine concentration in the skeletal muscle was significantly decreased after the endurance exercise and how taurine contributed to the exercise performance have been still unclear. We suggested that taurine in the skeletal muscles is utilized for NAT production and the NAT is excreted into the blood circulation during endurance exercises for the prevention of the accumulation of acetyl-CoA derived from acetate in the mitochondria.

Thus, we hypothesized that both taurine and carnitine may play roles in the regulations of the muscular mitochondrial amount of acetyl-CoA derived from short- and long-chain fatty acids in endurance exercises. The present study proposed the evaluation of the blood levels of NAT and ACT in humans before and after endurance exercises, and, furthermore, the investigation of the regulatory roles of taurine and carnitine in the mitochondrial balance of acetyl-CoA and free-CoA via the production of NAT and ACT using cultured skeletal muscle cells derived from humans.

## 2. Results

### 2.1. Serum Concentrations of Taurine, Carnitine, Its Acetylated Forms, and Energy Substrates during Endurace Exercises

**Figure 2:**
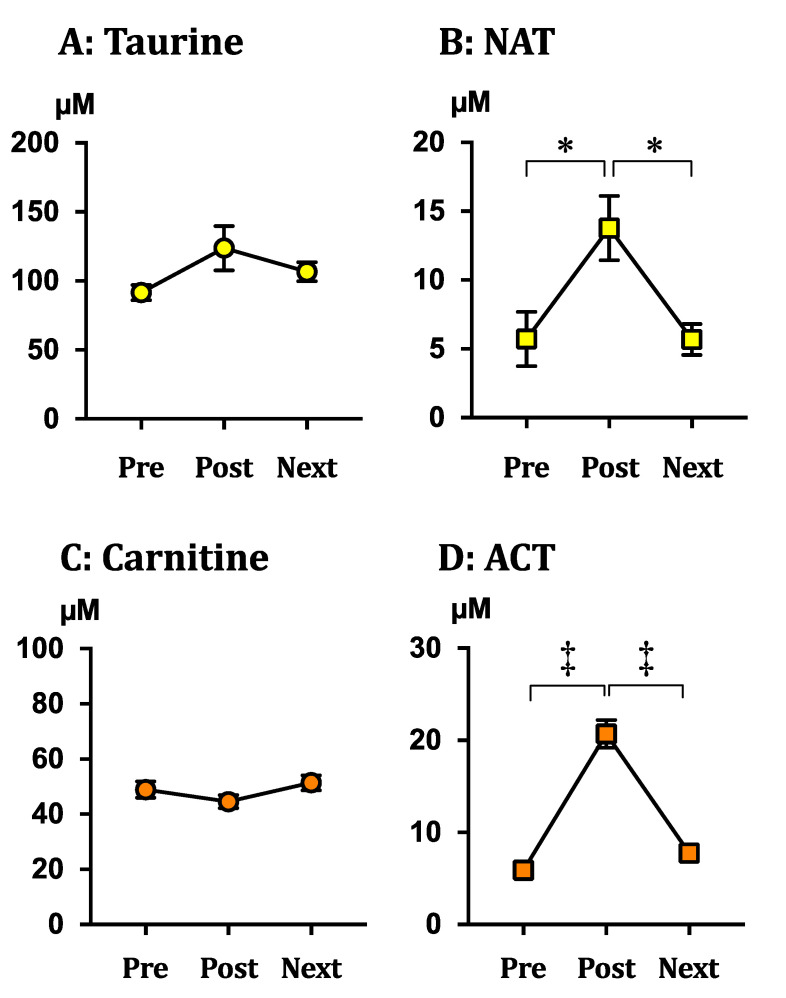
Serum levels of taurine (**A**), carnitine (**C**), and its acetylated forms, NAT (**B**) and ACT (**D**), before and after the endurance exercise. Serum was collected 1 day before (Pre), immediately after (Post), and the day after (Next) the full-marathon race. Taurine, NAT, carnitine, and ACT were simultaneously measured using an HPLC-MS/MS system. The data shown refer to the mean ± SEM. * *p* < 0.05 and 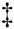 *p* < 0.0001 were analyzed by one-way ANOVA post-hoc tests. Abbreviations: NAT; *N*-acetyltaurine, ACT; acetylcarnitine.

Serum concentrations of taurine, NAT, carnitine, ACT, and other energy substrates were evaluated one day before (Pre), immediately after (Post), and the day after (Next) a full-marathon race. The taurine concentration was slightly higher in Post, but there was no significant difference among the three points (Figure 2A). On the contrary, the NAT concentration was significantly increased in Post compared to that in Pre, and the concentration in Next decreased significantly to the level in Pre (Figure 2B).

Similar to taurine concentration, there was no significant change in serum carnitine concentration before and after the exercise (Figure 2C). Serum ACT concentration was markedly and significantly higher in Post than in Pre, and it in Next, significantly decreased to the level in Pre (Figure 2D).

Serum concentrations of lactate, free fatty acid (FFA), and β-hydroxybutyrate significantly increased in Post compared to those in Pre (Table 1). On the next day, these energy-related parameters recovered to the level in Pre. There were no significant differences in serum glucose concentration among the three points (Table 1).

### 2.2. Intracellular and Extracellular Taurine, NAT, Carnitine, and ACT Concentrations in the Myotube Exposed to Acetate Combined with Taurine or Carnitine

To investigate whether the NAT and ACT are generated in the skeletal muscle cells by reaction with acetate and excreted to extracellular fluid, the myotube differentiated from human primary myoblast was exposed to sodium acetate following taurine or carnitine treatments, and then the NAT and ACT levels in the culture medium as well as in the cells were measured.

**Figure 3:**
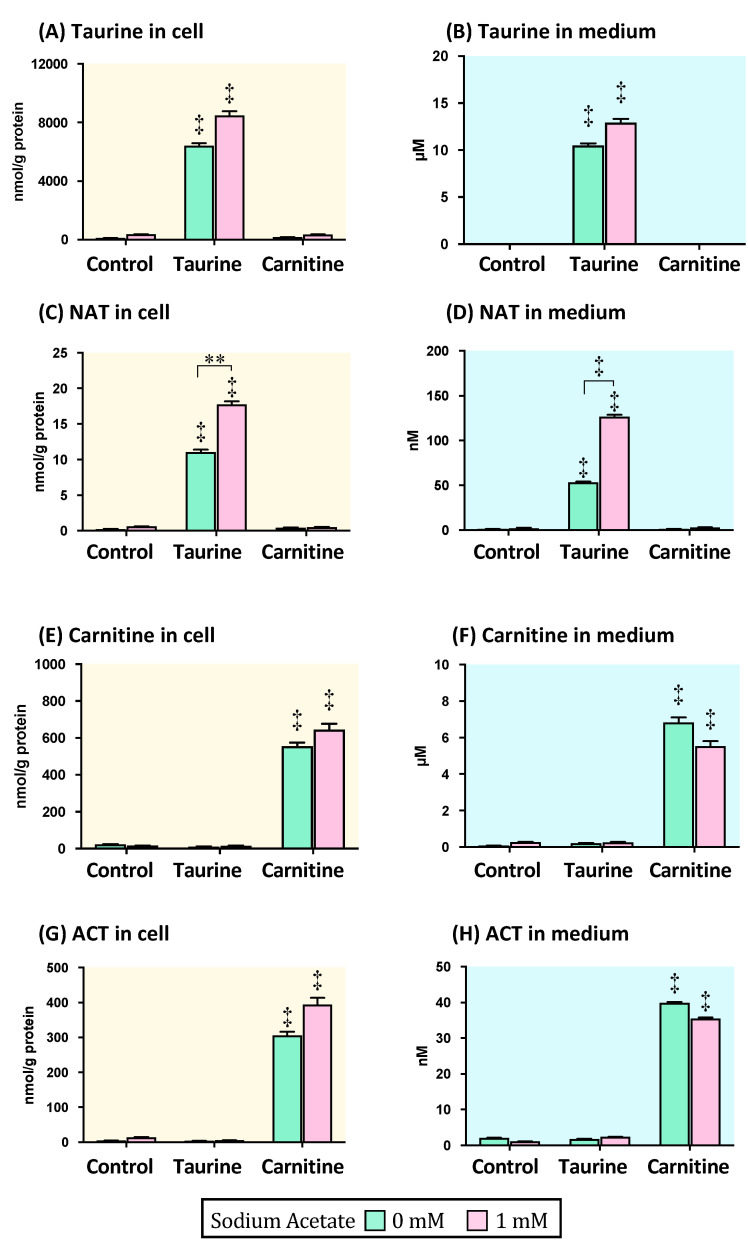
Taurine, carnitine, and its acetylated forms (NAT and ACT) in the myotube cell and culture medium after the treatments of taurine, carnitine, and acetate. The myotubes were treated with either 20 mM taurine or 300 µM carnitine for 24 h, and then exposed to 1 mM sodium acetate for 24 h. Thereafter, taurine, NAT, carnitine, and ACT concentrations in the cell (**A**,**C**,**E**,**G**) and culture medium (**B**,**D**,**F**,**H**) were measured. The data shown refer to the mean ± SEM. ** *p* < 0.01 and 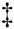 *p* < 0.0001 show the significant differences analyzed by the two-way ANOVA multiple comparison test. The symbols in the columns without bars show the significant difference compared to the respective control without taurine or carnitine treatment. Abbreviations: NAT; *N*-acetyltaurine, ACT; acetylcarnitine.

The myotube was exposed to 1 mM sodium acetate for 24 h following the pretreatments with 2 mM taurine or 300 µM carnitine for 24 h. Taurine level in the cell and culture medium was significantly increased by taurine treatment regardless of the presence of acetate (Figure 3A,B), while there was no significant difference for the exposure to acetate in the pretreatments of taurine and carnitine (Figure 3A,B). In both cell and culture medium, NAT concentration significantly increased under the taurine pretreatment conditions regardless of the presence of acetate compared to that under the respective untreated control condition (Figure 3C,D); however, under the taurine pretreatment condition, the concentration was significantly higher with acetate exposure than without (Figure 3C,D).

Carnitine and ACT concentrations in the cell and culture medium were significantly higher for the carnitine pretreatment than for the untreated control and taurine pretreatment, regardless of the exposure to acetate (Figure 3E–H). There were no significant differences in the carnitine and ACT concentrations of both the cell and culture mediums regardless of the presence of acetate (Figure 3E–H).

### 2.3. Extracellular NAT and ACT Concentrations in the Myotube Exposed to Palmitic Acid Combined with Taurine or/and Carnitine

In the next experiment, the myotube was used to investigate whether the NAT and ACT were generated in the skeletal muscle cells in the β-oxidation reaction and excreted to the extracellular fluid. The myotube was exposed to fatty acid following taurine or carnitine treatments, and then NAT and ACT levels in the culture medium were measured.

**Figure 4:**
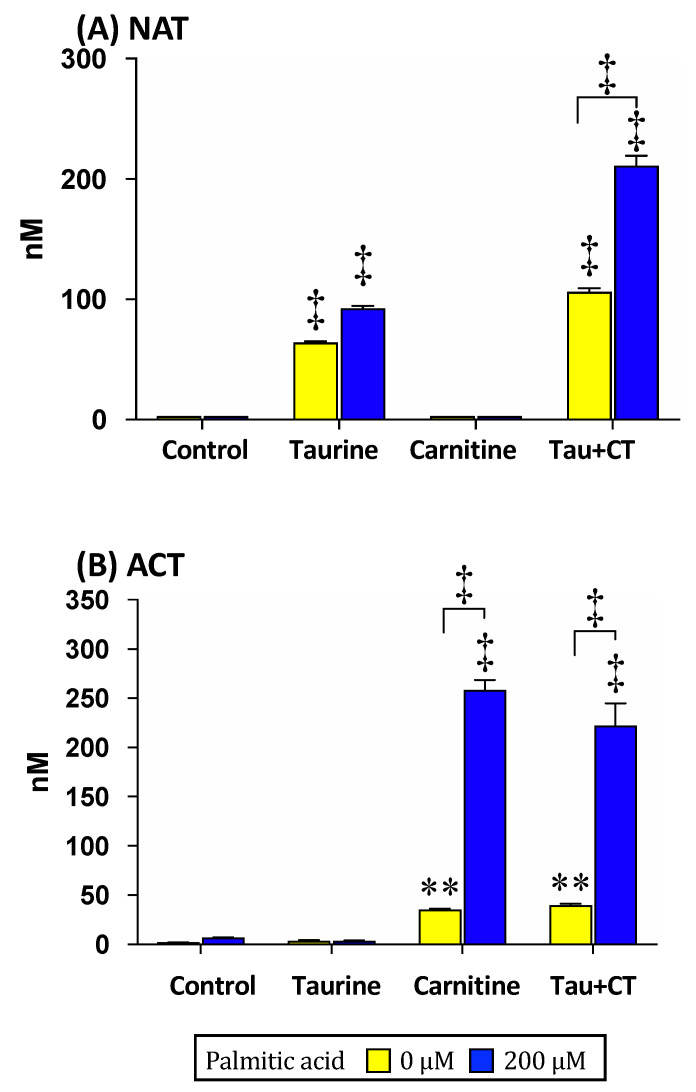
NAT and ACT concentrations in the culture medium of the myotube after exposure to palmitic acid combined with taurine and/or carnitine. The myotube was treated with 20 mM taurine or/and 300 µM carnitine for 24 h. Then, it was exposed to 200 µM palmitic acid for 24 h. NAT (**A**) and ACT (**B**) concentrations in the culture medium were measured. The data shown refer to the mean ± SEM. ** *p* < 0.01 and 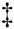 *p* < 0.0001 show the significant differences analyzed by the two-way ANOVA multiple comparison test. The symbols on the columns without bars show the significant difference compared to those in the non-pretreated control and taurine pretreated with palmitic acid. Abbreviations: NAT; *N*-acetyltaurine, ACT; acetylcarnitine, Tau+CT; taurine and carnitine.

In the culture medium, NAT and ACT concentrations were measured after the exposure to 200 µM palmitic acid for 24 h following the pretreatments of 2 mM taurine and/or 50 or 300 µM carnitine for 24 h. NAT concentration significantly increased under the taurine pretreatment conditions (taurine alone and the combination with carnitine) regardless of the exposure to palmitic acid compared to that in the respective untreated control (Figure 4A). Particularly, in the combination of taurine and carnitine, the NAT concentration was significantly higher with palmitic acid exposure than without. There was no difference in NAT concentration of the pretreatment with taurine alone regardless of the exposure to palmitic acid. Under the conditions without taurine pretreatment (control and carnitine pretreatments), NAT in the culture medium was almost nondetectable.

On the contrary, ACT concentration significantly increased via the treatments with carnitine alone and together with taurine under the exposure to palmitic acid compared to those under the respective pretreatment conditions without palmitic acid exposure, with the untreated control, and with taurine and palmitic acid (Figure 4B). Under the condition with no palmitic acid exposure, ACT concentration was also significantly increased in the carnitine pretreatments (carnitine alone and combined with taurine) compared to that in the respective nontreatment.

### 2.4. Mitochondrial Acetyl-CoA/Free-CoA Ratio in the Myotube Exposed to Acetate or Palmitic Acid Combined with Taurine or Carnitine

For evaluations of the effects of taurine and carnitine on the mitochondrial acetyl-CoA/free-CoA ratio, the myotube was treated with 2 mM taurine or carnitine (50 or 300 µM) with the mitochondrial acetyl-CoA generative condition of 1 mM sodium acetate exposure or with the β-oxidation condition (200 µM palmitic acid with 50 µM carnitine).

**Figure 5:**
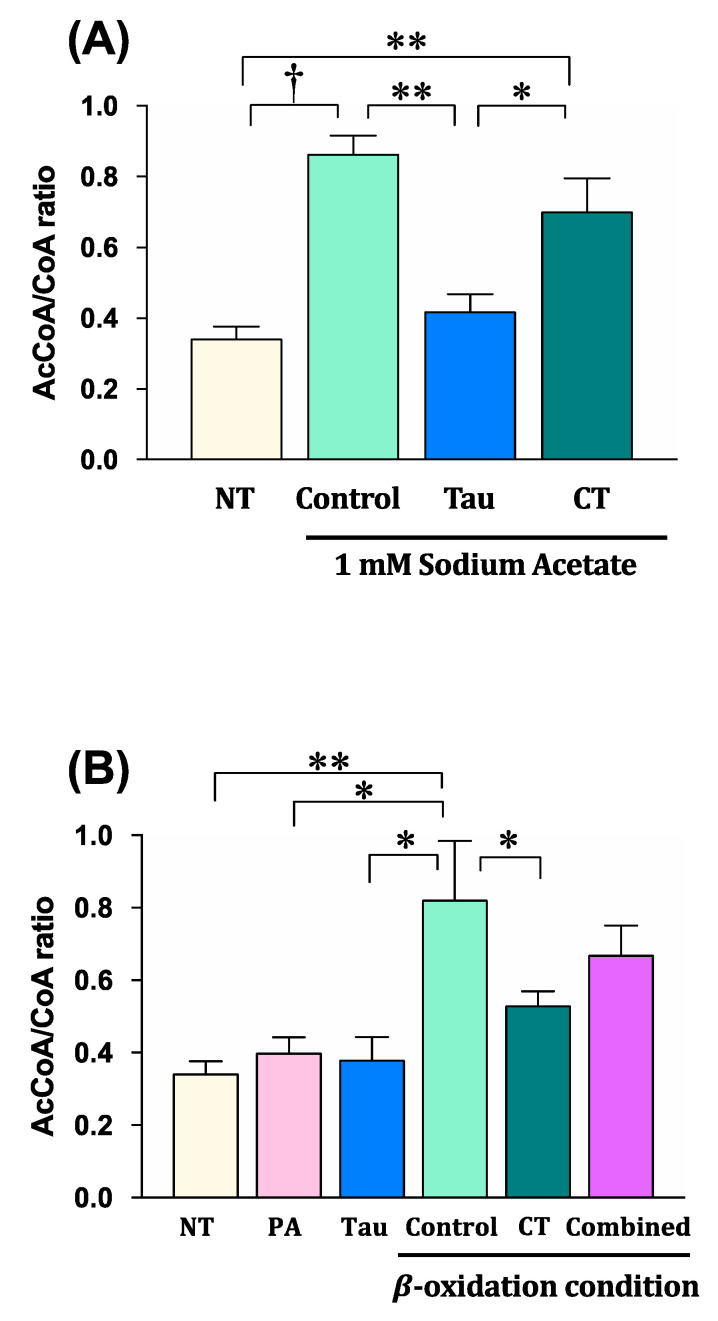
Mitochondrial acetyl-CoA/free-CoA ratio in the myotube exposed to acetate (**A**) and fatty acid (**B**) with taurine or/and carnitine treatments. (**A**) The myotube was exposed to 1 mM sodium acetate with 2 mM taurine or 300 µM carnitine for 24 h. Abbreviations: NT; no treatments with acetate, taurine, or carnitine, Control; 1 mM sodium acetate alone, Tau; 2 mM taurine with 1 mM sodium acetate, CT; 300 µM carnitine with 1 mM sodium acetate. (**B**) The myotube was exposed to 200 µM palmitic acid with 2 mM taurine, 50 µM carnitine, 300 µM carnitine, or a combination of 2 mM taurine and 50 µM carnitine for 24 h. Abbreviations: NT; no treatments with palmitic acid, taurine, or carntine, PA; 200 µM palmitic acid alone, Tau; 2 mM taurine with 200 µM palmitic acid, Control; 50 µM carnitine with 200 µM palmitic acid, CT; 300 µM carnitine with 200 µM palmitic acid, Combined; combination of 50 µM carnitine and 2 mM taurine with 200 µM palmitic acid, β-oxidation condition; culture condition with carnitine and palmitic acid. After these exposures for 24 h, acetyl-CoA and free CoA levels in the mitochondria were measured, and the ratio of acetyl-CoA to free CoA was calculated. The data shown refer to the mean ± SEM. * *p* < 0.05, ** *p* < 0.01, and 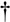 *p* < 0.001 show the significant differences analyzed by the one-way ANOVA post-hoc multiple comparison test.

Figure 5A shows the mitochondrial acetyl-CoA/free-CoA ratio under the sodium acetate exposure condition. The acetyl-CoA/free-CoA ratio significantly increased upon exposure to sodium acetate alone (Control) compared to that under the untreated condition without sodium acetate, taurine, or carnitine (NT). In the taurine treatment with acetate (Tau), the ratio did not increase, and it was maintained at the same level under the NT condition. On the contrary, the ratio significantly increased in the carnitine treatment with acetate (CT) compared to that under the NT and Tau conditions.

Figure 5B demonstrates the mitochondrial acetyl-CoA/free-CoA ratio in the fatty acid β-oxidation condition. The ratio significantly increased under the β-oxidation control condition (Control), which was the cotreatment with 200 µM palmitic acid and 50 µM carnitine, compared to those under the no treatment condition (NT), palmitic acid alone (PA), and combined with taurine (Tau). The result in the Control implies that the β-oxidation reaction progressed in the presence of carnitine and palmitic acid. There was no difference in the ratio among the carnitine untreated conditions (NT, PA, and Tau). On the contrary, the higher dose (300 µM) of carnitine with palmitic acid (CT) significantly suppressed the increase in ratio observed with the Control condition, while the taurine cotreatment with 50 µM carnitine (Combined) was lower, but not significantly, than in the Control.

## 3. Discussion

The present study evaluated the hypothesis that taurine and carnitine act as regulators of the mitochondrial acetyl-CoA/free-CoA ratio in the energy production from short- and long-chain fatty acids, via NAT and ACT formations in the skeletal muscles during endurance exercises. The results indicate that the serum levels of NAT and ACT significantly increased immediately after the full-marathon race. In the experiments of cultured skeletal muscle cells, the NAT was significantly excreted from the myotube to the culture medium when taurine was treated together with acetate, while significant ACT excretion was observed in the cotreatment of carnitine and palmitic acid. In addition, the significantly increased mitochondrial acetyl-CoA/free-CoA ratio seen for the treatments with acetate or fatty acid was significantly suppressed by cotreatments with taurine or carnitine, respectively.

Carnitine acts as the modulator of the accumulation of mitochondrial acetyl-CoA induced in fatty acid β-oxidation, excreting the consequent metabolite ACT from the skeletal muscles [1,2]. As fatty acid β-oxidation is activated to fulfill the high demand of energy production in the skeletal muscles during endurance exercises, we suggested in this study that the ACT concentration in blood elevates during the endurance exercises. In the results, serum concentrations of ACT, as well as β-hydroxybutyrate, significantly increased after the full-marathon race (Figure 2D and Table 1). The blood level of β-hydroxybutyrate, which is the most stable ketone body in blood, is established as a marker of hepatic fatty acid β-oxidation because the metabolite of fatty acid β-oxidation is excreted from the liver under ketogenic conditions such as during endurance exercises and starvation [22,23]. On the contrary, there are few studies in humans that illustrate the change in the blood ACT level during endurance exercises [24], while significant increases in the ACT levels in the skeletal muscles during exercise have been reported [1,25,26,27]. The formation of ACT in the skeletal muscles has been recognized as the marker for intramuscular ATP production via oxidative phosphorylation from glycolysis and fatty acid β-oxidation [26]. Based on the facts that most of the carnitine in the body is contained in the skeletal muscles [28] and carnitine is the essential regulator of the β-oxidation of skeletal muscles [29], the increased level of blood ACT is thought to reflect the increase in muscular β-oxidation activity in endurance exercises. This is also supported by the present results that show that ACT was excreted from the muscle cells exposed to the fatty acid together with carnitine (Figure 4B), along with the improvement in the mitochondrial acetyl-CoA/free-CoA ratio (Figure 5B).

Similar to ACT, a significantly increased NAT concentration in the serum was observed after the full-marathon race (Figure 2B). NAT has been reported to be a metabolite of taurine reacting with acetate, which is a product in the detoxification of ethanol in the liver, in order to facilitate the urinary excretion of excess acetate by increasing the hydrophilicities via reaction with taurine [9]. Acetate is also generated from acetyl-CoA in fatty acid β-oxidation, along with the ketone bodies in the liver under ketogenic conditions, and it is supplied as an energy fuel to extrahepatic tissues, including skeletal muscle. As acetate is metabolized directly back to the mitochondrial acetyl-CoA in one metabolic step by ACS2 (Figure 1) [5,6,7,8], acetate is thought to be a more efficient energy fuel than other nutrients, including glucose and fatty acids, which require multiple metabolic reactions to generate acetyl-CoA in skeletal muscles. However, it is possible for acetyl-CoA to accumulate in the mitochondria of skeletal muscle if acetate is excessively supplied from the liver during and after endurance exercises.

In addition to the abundance of taurine in the skeletal muscles [10,11,12] and the excretion of NAT from the skeletal muscle cells cultured with acetate and taurine, our previous study confirmed that plasma NAT concentration was increased, correlated with that in the skeletal muscle, but not in the liver, after a treadmill exercise in rats [30]. These findings strongly supported that the significantly increased NAT in serum was derived from the skeletal muscle during the endurance exercise in humans. Therefore, there is a possibility that the blood levels of NAT could be a potential biomarker of energy production status from short-chain fatty acids, similarly to ACT for long-chain fatty acids, during endurance exercise in the skeletal muscles of humans.

In the cell culture experiment, the acetyl-CoA/free CoA ratio in the mitochondria significantly increased upon acetate treatment (Figure 5A). Serum acetate concentration was likely to increase significantly along with the significant increase of β-hydroxybutyrate and FFA after the endurance exercises (Figure 2). Therefore, it is suggested that the mitochondrial acetyl-CoA derived from acetate might have increased in the skeletal muscles after the full-marathon race. The present cell culture experiments also showed that the significantly increased acetyl-CoA/free-CoA ratio in the mitochondria of the myotube was suppressed by taurine treatment, and that the excretion of NAT to the culture medium significantly increased (Figure 3D). Indeed, the serum NAT concentration significantly increased after the full-marathon race, and we preliminarily reported the significant increase in urinary NAT excretion in a human after endurance exercise [31]. Our data support the hypothesis that taurine acts as a buffer of the acetyl moiety of excess mitochondrial acetyl-CoA derived from acetate in the skeletal muscles during endurance exercises.

In the myotube experiments, NAT concentrations in both cell and culture medium were significantly higher in the taurine and acetate cotreatment than in the treatment with taurine alone (Figure 3C,D). On the contrary, ACT concentrations in both cell and culture mediums were significantly increased via carnitine treatment (Figure 3G,H), but the excretion level was not influenced in the presence of acetate. Thus, these results imply that ACT is not generated from the acetyl-CoA that is derived from acetate. In addition, the NAT excretion to the culture medium significantly increased via taurine treatment under the β-oxidation condition (Figure 4A), implying that taurine contributes to the regulation of the mitochondrial acetyl-CoA/free-CoA ratio in the fatty acid β-oxidation.

Together with the many physiological actions [13,14,15,16,17], including the enhancement of exercise performance [18,19,20], taurine may have beneficial effects against physical fatigue and recovery by exercise. The present study shows that taurine plays a preventive role in the increase in the mitochondrial acetyl-CoA/free-CoA ratio derived from acetate in the skeletal muscles by the generation of NAT, similar to the role carnitine plays in β-oxidation, which results in ACT generation. The mitochondrial acetyl-CoA accumulation leads to the delay in the glycolysis pathway and insulin resistance in the skeletal muscles; the reduction in PDH activity by mitochondrial acetyl-CoA delays the metabolic flux from glycolysis to the TCA cycle [32], and the delayed flux reduces the phosphofructokinase activity and accumulates glucose-6-phosphate, which allosterically reduces the activity of hexokinase, which is the rate-limiting enzyme of glycolysis. This continuous negative feedback controlled by the cell energy state finally results in a decline in insulin-dependent glucose uptake [33,34,35,36]. In a previous study, we showed that insulin resistance was caused in the C2C12 myotube by excess ACT treatment that induces the acetyl-CoA accumulation in the mitochondria through the reverse transfer of the acetyl moiety to free-CoA [37]. Therefore, we speculated that the beneficial effects of taurine on fatigue and recovery after exercise is related to the improvement of the impaired glycolysis by the regulation of mitochondrial acetyl-CoA accumulation through NAT generation.

## 4. Materials and Methods

### 4.1. Materials

Taurine, L-carnitine, sodium acetate, acetic anhydride, sucrose, EDTA, potassium dihydrogen phosphate, acetonitrile, 2-propanol, methanol, 4-dimethylaminopyridine, and pyridine were purchased from FUJIFILM Wako Pure Chemical Corporation (Osaka, Japan). NAT was purchased from Carbosynth Limited (Berkshire, UK). Acetyl-L-carnitine HCl, dibutylammonium acetate (DBAA), 2-pyridinemethanol (2PM), and 2-methyl-6-nitrobenzoic anhydride were purchased from Tokyo Kasei Kogyo (Tokyo, Japan). [^2^H_4_]taurine (taurine-d4), acetyl-L-[^2^H_3_]carnitine HCl (ACT-d3), L-[^2^H_3_]carnitine HCl (carnitine-d3), and DL-lactate-[^2^H_3_] (lactate-d3) were obtained from C/D/N Isotopes Inc. (Quebec, Canada). NAT-d4 was synthesized from taurine-d4 by the reaction with acetic anhydride [38]. Taurine-d4, NAT-d4, carnitine-d3, and ACT-d3 were mixed with acetonitrile to use as an internal standard (IS) mixture. Acetyl-CoA, HMG-CoA, free-CoA, sodium [^13^C_4_]DL-β-hydroxybutyrate (βHB-^13^C_4_), and bovine serum albumin (BSA) were obtained from Merck, KGaA (Darmstadt, Germany). Human primary skeletal muscle myoblasts isolated from the rectus abdominus muscle, and growth medium (GM) and differentiation medium (DM) specialized for human cells were purchased from ZenBio, Inc. (Durham, UK). All of the other reagents for the cell culture experiments were purchased from Thermo Fisher Scientific (Gibco^®^, Waltham, MA, USA). The solvents used for analysis were of analytical grade.

### 4.2. Exercise Experiment in Humans

In the human study, 17 healthy male volunteers who participated in the 32nd Tsukuba full marathon (held in Ibaraki, Japan) were recruited. The average goal time of the subjects was 264.6 ± 60.6 min (mean ± standard error [SEM]). In the subjects, blood was collected at three points: One day before (Pre), immediately after (Post), and the day after (Next) the full-marathon race. The serum was maintained at −20 °C until analysis.

### 4.3. Differentiation of Cultured Skeletal Muscle Cell and Treatments

For the experiments of skeletal muscle cell culture, human primary skeletal muscle myoblasts were used followed by differentiation to myotubes according to the manufacture by ZenBio, Inc. The myoblasts seeded in a 12-well plate were cultured with the GM until confluent and were then differentiated to the myotube with the DM for 5 days. The cells were maintained at 37 °C in a humid atmosphere of 5% CO_2_.

For the acetate exposure experiment, the myotube was exposed to 20 mM taurine or 300 µM carnitine in the DM for 24 h. Doses of taurine and carnitine were referenced with our previous studies [37,39]. After washing with PBS twice, the myotube was further exposed to 1 mM sodium acetate in GM for an additional 24 h.

For the fatty acid exposure experiment, the myotube was exposed to 20 mM taurine and/or 300 µM carnitine in the DM for 24 h; then, it was exposed to 200 µM palmitic acid dissolved in a solution of 10% BSA-PBS in the DMEM including 1 g/L glucose without FBS for an additional 24 h.

After both experiments, the myotube and culture medium were collected for analyses of taurine, NAT, carnitine, ACT, free-CoA, and acetyl-CoA using an HPLC-MS/MS system. Data were obtained from four independent experiments (*n* = 4).

### 4.4. Taurine, NAT, Carnitine, and ACT Analyses

Taurine, NAT, carnitine, and ACT in samples were quantified by an HPLC-MS/MS system according to our previous study [37]. The myotube cells collected in a collection tube were sonicated in 500 µL of KP buffer (10 mM potassium-phosphate buffer, pH 5.5). After centrifugation at 3500× *g* at 4 °C for 10 min, the supernatant was used for analysis. The protein concentration in the supernatant was measured using a Pierce^®^ BCA Protein Assay Kit (Thermo Fischer Scientific Inc., Rockford, MI, USA). Fifty microliters of the culture medium and the supernatants from the cell, and 5 µL of human serum were mixed with 50 µL of IS solution containing 100 ng of taurine-d4, 1 ng of NAT-d4, 5 ng of carnitine-d3, and 5 ng of ACT-d3 in acetonitrile–water (19:1, *v*/*v*). After centrifugation at 2000× *g* for 1 min, the supernatant was evaporated to dryness at 80 °C under a nitrogen stream. The residue was redissolved in 60 µL of 0.1% formic acid, and an aliquot (5 µL) was injected into the LC-MS/MS system and was analyzed in electrospray ionization (ESI) mode.

Chromatographic separation was performed using a Hypersil GOLD aQ column (150 × 2.1 mm, 3 μm, Thermo Fischer Scientific Inc.) at 40 °C. The mobile phase was methanol–water (1:9, *v*/*v*) containing 0.1% formic acid, at a flow rate of 200 μL/min. The MS/MS conditions were spray voltage, 3000 V; vaporizer temperature, 450 °C; sheath gas (nitrogen) pressure, 50 psi; auxiliary gas (nitrogen) flow, 15 arbitrary units; ion transfer capillary temperature, 220 °C; collision gas (argon) pressure, 1.0 motor; collision energy, 20 V; ion polarity, positive; selected reaction monitoring (SRM), *m*/*z* 124 → *m*/*z* 80 for the taurine, *m*/*z* 128 → *m*/*z* 80 for the taurine-d4 variant, *m*/*z* 166 → *m*/z 80 for the NAT, *m*/*z* 170 → *m*/*z* 80 for the NAT-d4 variant, *m*/*z* 162 → *m*/*z* 103 for the carnitine, *m*/*z* 165 → *m*/*z* 103 for the carnitine-d3 variant, *m*/*z* 204 → *m*/*z* 85 for the ACT, and *m*/*z* 207 → *m*/*z* 85 for the ACT-d3 variant. The analyzed results for the cell sample are expressed per protein.

### 4.5. Other Blood Biochemical Analyses

In serum, glucose and FFA concentrations were measured using commercially available kits: Glucose CII-test Wako and NEFA C-test kit Wako (FUJIFILM Wako Pure Chemical Corporation).

Lactate and β-hydroxybutyrate were analyzed using the HPLC-MS/MS system with derivatization into 2PM esters according to our previous studies [22,40]. Serum (5 µL) was mixed with 200 µL of acetonitrile–water (19:1, *v*/*v*) containing 100 ng of βHB-^13^C_4_ and 500 ng of lactate-d3 as an IS. The supernatant obtained after centrifugation at 2000× *g* for 1 min was evaporated to dryness at 80 °C under a nitrogen stream. The residue was resolved with a 50 µL mixture of reagents containing 2-methyl-6-nitrobenzoic anhydride, 4-dimethylaminopyridine, pyridine, and 2-PM, and it was incubated at room temperature for 30 min. Next, the reactant was centrifugated at 700× *g* for 1 min followed by mixing with 1 mL of diethyl ether, and the supernatant was evaporated at 55 °C under a nitrogen stream. The residue redissolved in 100 μL of 0.1% formic acid–water (*v*/*v*), and 5 µL of the supernatant after spin down was injected into the HPLC-MS/MS system. Chromatographic separation was performed using the Hypersil GOLD aQ column at 40 °C. The mobile phase was initially the acetonitrile–water containing 0.2% formic acid at a flow rate of 300 µL/min for 5 min; then, the flow rate was switched to 300 μL/min for an additional 7 min. The general MS/MS conditions were as follows: Spray voltage, 3000 V; vaporizer temperature, 450 °C; sheath gas (nitrogen) pressure, 50 psi; auxiliary gas (nitrogen) flow, 15 arbitrary units; ion transfer capillary temperature, 220 °C; collision gas (argon) pressure, 1.0 mTorr; collision energy, 15 V; ion polarity, positive; SRM, *m*/*z* 196 → *m*/*z* 110 for the 2PM-β-hydroxybutyrate, *m*/*z* 200 → *m*/*z* 110 for the 2PM-βHB-^13^C_4_, *m*/*z* 182.1 → *m*/*z* 110.1 for the 2PM-Lactate, and *m*/*z* 185.1 → *m*/*z* 110.1 for the 2PM-Lactate-d3.

### 4.6. Mitochondrial Free-CoA and Acetyl-CoA Concentrations in Myotubes

The mitochondrial fraction was prepared from the collected myotubes according to the previously reported method [41], and acetyl-CoA and free-CoA contents were measured by our method reported previously [37]. The myotube was homogenized with a loose-fitting Teflon pestle in 4 volumes of 3 mM Tris-HCl buffer (pH 7.4) containing 0.25 mM sucrose and 0.1 mM EDTA, and it was centrifuged at 700× *g* for 10 min. The supernatant was further centrifuged at 7000× *g* for 20 min. After the centrifugation, the mitochondrial pellet was homogenized in 100 μL of freshly prepared potassium dihydrogen phosphate (pH 4.9) (100 mM) and 100 μL of acetonitrile, 2-propanol, and methanol (3:1:1, *v*/*v*/*v*) with 50 pmol of the HMG-CoA as an IS; then, it was centrifuged at 16,000× *g* at 4 °C for 10 min. The supernatant was dried at 50 °C under a nitrogen stream and was then resuspended in 50 μL of 50% methanol–water solution. After centrifugation at 14,000× *g* for 10 min at 4 °C, 5 µL of the supernatant was injected into the LC-MS/MS system and was analyzed in ESI mode for quantification of the mitochondrial acetyl-CoA and free-CoA.

Chromatographic separation was performed using the Hypersil GOLD aQ column at 40 °C, with the following gradient system at a flow rate of 200 μL/min. Initially, the mobile phase comprised 5 mM aqueous DBAA-methanol (4:1, *v*/*v*), which was programmed to change to 5 mM aqueous DBAA-methanol (3:7, *v*/*v*) in a linear manner over 10 min. The MS/MS conditions were spray voltage, 2500 V; vaporizer temperature, 450 °C; sheath gas (nitrogen) pressure, 50 psi; auxiliary gas (nitrogen) flow, 15 arbitrary units; ion transfer capillary temperature, 220 °C; collision gas (argon) pressure, 1.0 motor; collision energy, 35 V; ion polarity, negative; SRM, *m*/*z* 766 → *m*/*z* 408, *m*/*z* 808 → *m*/*z* 408, and *m*/*z* 910.1 → *m*/*z* 408.1 for CoA, acetyl-CoA, and HMG-CoA, respectively. The ratio of acetyl-CoA to free-CoA was calculated from the value of mitochondrial acetyl-CoA and free-CoA contents.

### 4.7. Statistical Analysis

Statistical significance was determined via the unpaired Student’s *t*-test, or one-way or two-way ANOVA multiple comparison tests. The data expressed refer to the mean ± SEM. Differences were considered statistically significant when the calculated *p* value was less than 0.05.

## 5. Conclusions

The present study confirmed that the serum NAT and ACT concentrations significantly increased after endurance exercises. In the cultured muscle cells, the increased mitochondrial acetyl-CoA/free-CoA ratios induced by acetate or palmitic acid exposures were significantly suppressed via taurine or carnitine treatment, respectively, together with the increase in extracellular NAT and ACT excretions. In conclusion, the present results support the hypothesis that taurine and carnitine act as regulators of the acetyl moiety of AcCoA derived from short- and long-chain fatty acids in the mitochondria of skeletal muscles during endurance exercises by excretions of NAT and ACT to blood from the skeletal muscle. In turn, the NAT and ACT production may support the regulation of energy metabolism during exercise and of recovery from muscle fatigue after endurance exercises. The present findings suggest that blood NAT and ACT levels could be the parameters of energy production status from acetate and fatty acids in the skeletal muscles in endurance exercise.

## Figures and Tables

**Table 1:** Serum levels of biochemical energy-related parameters before and after the exercises.

Parameter	Pre	Post	Next
Glucose (mg/dL)	86.5 ± 1.0	83.0 ± 3.0	81.0 ± 1.7
Lactate (mg/dL)	18.1 ± 1.5	43.3 ± 3.9 *aa*	20.2 ± 1.0 *bb*
FFA (mEq/mL)	0.59 ± 0.08	1.73 ± 0.12 *aa*	0.64 ± 0.07 *bb*
β-hydroxybutyrate (µM)	48.2 ± 13.8	305.6 ± 40.1 *aa*	74.8 ± 15.6 *b*

Footnote: The data shown refer to the mean ± SEM. *aa*; *p* < 0.0001 compared to the Pre, *b*, and *bb*; *p* < 0.001 and *p* < 0.0001 compared to the Post by one-way ANOVA post-hoc multiple-comparison test. Abbreviations: FFA; free fatty acid, Pre; before the full marathon, Post; immediately after the full marathon, Next; the day after the full marathon.

## Data Availability

Not applicable.

## Abbreviations

The following abbreviations are used in this manuscript:

βHB-^13^C4	[^13^C_4_]β-hydroxybutyrate-^13^C4
2PM	2-pyridinemethanol
ACT	acetylcarnitine
ACT-d3	acetyl-L-[^2^H_3_]carnitine HCl
ACS2	acetyl-CoA synthetase 2
BSA	bovine serum albumin
CACT	carnitine acylcarnitine translocase
carnitine-d3	L-[^2^H_3_]carnitine HCl
CAT	carnitine acetyltransferase
CPT	carnitine palmitoyl transferase
CrAT	carnitine acetyltransferase
CT	carnitine
DBAA	dibutylammonium acetate
DM	differentiation medium
DMEM	Dulbecco’s modified Eagle’s medium
ESI	electrospray ionization
FBS	Fetal bovine serum
FFA	free fatty acid
GLUT4	glucose transporter 4
GM	growth medium
IS	internal standard
lactate-d3	DL-lactate-[^2^H_3_]
MCT	monocarboxylate transporter
NAT	*N*-acetyltaurine
OCTN2	organic anion transporter 2
PDH	pyruvate dehydrogenase
SEM	standard error
taurine-d4	[^2^H_4_]taurine
